# Fast and slow errors: Logistic regression to identify patterns in accuracy–response time relationships

**DOI:** 10.3758/s13428-018-1110-z

**Published:** 2018-09-05

**Authors:** Leendert van Maanen, Dimitris Katsimpokis, A. Dilene van Campen

**Affiliations:** 1 Department of Psychology, University of Amsterdam, P.O. Box 15906, 1001 NK Amsterdam, Netherlands; 2 Donders Center for Brain and Cognition, Radboud University, Nijmegen, Netherlands

**Keywords:** Response times, Accuracy, Conditional accuracy function

## Abstract

Understanding error and response time patterns is essential for making inferences in several domains of cognitive psychology. Crucial insights on cognitive performance and typical behavioral patterns are disclosed by using distributional analyses such as *conditional accuracy functions (CAFs)* instead of mean statistics. Several common behavioral error patterns revealed by CAFs are frequently described in the literature: response capture (associated with relatively fast errors), time pressure or urgency paradigms (slow errors), or cue-induced speed–accuracy trade-off (evenly distributed errors). Unfortunately, the standard way of computing CAFs is problematic, because accuracy is averaged in RT bins. Here we present a novel way of analyzing accuracy–RT relationships on the basis of nonlinear logistic regression, to handle these problematic aspects of RT binning. First we evaluate the parametric robustness of the logistic regression CAF through parameter recovery. Second, we apply the function to three existing data sets showing that specific parametric changes in the logistic regression CAF can consistently describe common behavioral patterns (such as response capture, time pressure, and speed–accuracy trade-off). Finally, we discuss potential modifications for future research.

In many domains of cognitive psychology, understanding the different types of error and response time (RT) patterns is necessary for appropriate inferences. Consider, for example, one of the hallmark experimental paradigms of the cognitive control literature, the Simon task (Van Campen, Keuken, Van den Wildenberg, & Ridderinkhof, 2014; Van Maanen, Turner, & Forstmann, 2015). In this task, participants are asked to respond to some relevant stimulus feature with either their left or their right hand. Crucially, the stimulus is placed on the left or right side of a computer screen, creating a congruency or incongruency between the stimulus location and the response hand. On average, incongruent stimulus–response mappings result in relatively slower and more incorrect responses than do congruent stimulus–response mappings, suggesting a relatively simple mechanism. Interestingly, however, analyzing the full RT distributions of congruent and incongruent mappings for RT and error patterns resulted in a large body of research acknowledging the existence of a more complex picture of different underlying processes within the Simon task (Burle, Possamaï, Vidal, Bonnet, & Hasbroucq, 2002; De Jong, Liang, & Lauber, 1994; Forstmann et al., 2008; Hommel, 1993, 1994; Proctor, Miles, & Baroni, 2011; Ridderinkhof, 2002; Stürmer, Leuthold, Soetens, Schröter, & Sommer, 2002; Tagliabue, Zorzi, Umiltà, & Bassignani, 2000; Van Campen, Kunert, Van den Wildenberg, & Ridderinkhof, 2018; Van Campen et al., 2014; Van den Wildenberg et al., 2010).

One popular analysis tool that jointly considers the accuracy of responses and the distribution of their RTs is the *conditional accuracy function* (CAF; Gratton, Coles, Sirevaag, Eriksen, & Donchin, 1988; Heitz, 2014; Lappin & Disch, 1972; Proctor et al., 2011; Ratcliff, 1979; Ridderinkhof, 2002). A CAF expresses how the accuracy of responses depends on the speed of responses, by formulating how the probability of a correct response depends on the RT.

Analyzing CAFs is appealing because it allows one to investigate the categorical relationships between responses (typically, correct or incorrect) and RTs. The first type of categorical error–RT relationship that is often observed is that the RTs of incorrect responses are relatively fast. This is for example the case in the previously introduced Simon paradigm, in which a higher proportion of these fast errors for incongruent stimulus–response mappings than congruent stimulus–response mappings is interpreted as “response capture,” the automatic activation of the (incorrect) response hand invoked by the stimulus location (Forstmann et al., 2008; Ridderinkhof, 2002; Ulrich, Schroter, Leuthold, & Birngruber, 2015; Van Campen et al., 2014; Van Campen et al., 2018; Van den Wildenberg et al., 2010; Van Wouwe et al., 2016).

A second common categorical error–RT relationship entails response times of incorrect responses that are slower than average. In certain experimental settings, this is sometimes interpreted as an indication of time pressure or urgency on behavior. Time pressure would result in a higher proportion of relatively late incorrect responses, representing that as participants “feel” the time pressure, they start to make errors (Hanks, Kiani, & Shadlen, 2014; Murphy, Boonstra, & Nieuwenhuis, 2016; Thura & Cisek, 2016).

A third relationship between accuracy and RTs is that erroneous responses are distributed evenly across the RT distribution, and there is in fact no dependence of the correctness of the response on RT (Donkin & Van Maanen, 2014; Mulder & Van Maanen, 2013; Van Ede, de Lange, & Maris, 2012). However, the proportion of errors might still depend on some experimental manipulation, such as a cue-induced speed–accuracy trade-off task. In such an experiment, participants are instructed to focus either on accurately responding (ignoring response speed) or on response speed. The focus on response speed typically comes at the cost of making more errors, which are evenly distributed across the RT distribution (Heitz, 2014; Schouten & Bekker, 1967; Van Maanen et al., 2011; Wickelgren, 1977). Theoretical models of this kind of speed–accuracy trade-off behavior propose that people confronted with such a trade-off entertain a critical confidence value (threshold setting in sequential-sampling models; e.g., Bogacz, Wagenmakers, Forstmann, & Nieuwenhuis, 2010), which triggers a response as soon as that critical value is surpassed. This typically results in an equal distribution of errors across the RT distribution, even though accuracy and RT are both affected by changing the critical confidence value.

To draw inferences such as the ones sketched out above, CAFs have been extensively used. The standard method for quantifying CAFs is to indicate a set of RT bins, and then to compute the average proportion of correct responses per RT bin. However, there is not a generally accepted method for computing RT bins. Hyndman and Fan (1996) discussed nine different methods that are used in various statistical packages. In addition, the *number* of RT bins is also a matter of judgment. In the cognitive control literature, often three or four bins are used (Forstmann et al., 2008; Van den Wildenberg et al., 2010), but in many domains, dividing the RT distribution into five or six bins is much more common (Brown & Heathcote, 2008; Heathcote, Brown, & Mewhort, 2002).

The choice of the number of bins may have important consequences for the potential conclusions drawn from a particular data set. This is illustrated in Fig. 1, in which we present simulated data from a task in which fast errors occur (Fig. 1a) and from a task in which slow errors occur (Fig. 1b). The black dots in Fig. 1, with an accuracy of 0 or 1, represent the responses in a fictional experiment. The locations on the *x*-axis indicate the associated RTs. In Fig. 1a, errors (with an accuracy of 0) are relatively fast, which is visible by the number of error responses on the left side of the *x*-axis. In Fig. 1b, errors are slow, indicated by their high relative frequency, on the right side of the *x*-axis (as well as by the lower frequency of correct responses—accuracy of 1—on the right side). Each panel shows three CAFs (red, blue, and green), computed by averaging the error proportion for a particular RT bin. The numbers of bins are 10 (red), 7 (blue), and 4 (green). If errors are predominantly associated with the fastest responses (Fig. 1a), then the proportion of errors in the first RT bin depends on the width of that bin. If the bin is wider, reflecting a larger part of the RT distribution, then the error proportion will decrease accordingly. In Fig. 1a, this is illustrated as the thicker part of each CAF.

**Fig. 1:**
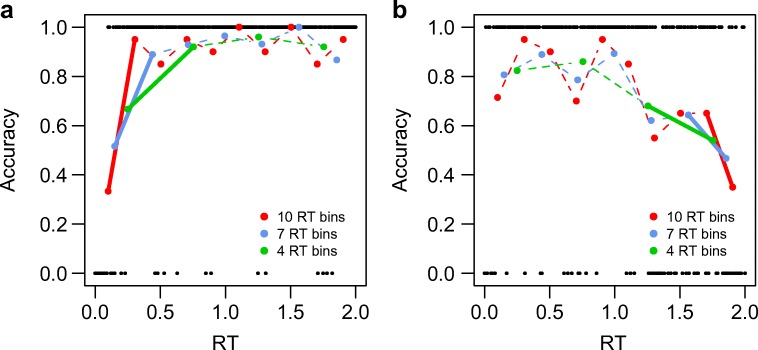
Two examples in which the number of response time (RT) bins is a potential threat for statistical inference. (**a**) The data contain a proportion of fast incorrect responses, indicated by the larger number of errors (data points) on the left of the RT scale, relative to the right. The estimated proportion of fast errors (solid line segments of the CAFs) depends on the number of bins in the analysis. (**b**) Decline in accuracy with RT, as measured by the accuracy difference between the last two bins. The reliability of this measure depends critically on the chosen number of bins and presents a potential threat for statistical interference.

If errors are predominantly associated with slower responses, then the number of bins influences the measurement of the change in error proportion. In Fig. 1b, we have highlighted this in the last segment of each CAF. Also, because there are fewer responses per RT bin, the uncertainty in these measurements increases.

In summary, the effect of the chosen number of bins—and related to this, the width of the bin, as illustrated in Fig. 1—is the first potential pitfall for applying CAFs. The reliability of the estimate of the error rate within a bin depends critically on the chosen number of bins and presents a potential threat for statistical interferences.

The second potential pitfall is related to the way the size of the RT bins is determined. The typical approach is to set the boundaries of each RT bin on the basis of the frequency of responses per bin (i.e., using the quantiles of the RT distribution; sometimes the range of the RT distribution is used, to ensure that the ranges of the RT bins remain equal instead of the frequency of observations). If one wants to draw a conclusion about a difference between conditions, however, the RT bins can be determined using the quantiles of each condition separately or in combination with all others. Which method is chosen may considerably impact the conclusion. For example, if the RT distributions of two conditions are shifted relative to each other, but the bin sizes are determined according to the combined RT distribution, then the proportions of fast errors may seem to differ just because the number of trials from each condition differs per RT bin.

Finally, the third pitfall is that averaging accuracy over RT bins raises statistical problems with respect to hypothesis testing. The response variable is treated as continuous on the interval [0, 1], rather than categorical with two levels (correct or incorrect response, often coded as 0 or 1). Treating categorical variables as continuous is problematic from a statistical viewpoint (Jaeger, 2008). For example, the confidence intervals on the expected proportion of correct responses for a particular RT bin could exceed the levels of the categorical variable (e.g., a confidence interval of CI = [0.9, 1.03], where the categorical levels are 0 and 1, exceeds the real maximum possible score). Furthermore, the general linear model assumes equal variances across groups. As Jaeger pointed out, this is not the case for categorical variables with two levels that are binomially distributed, because by definition in binomial distributions, a change in the variable’s mean induces a change in its variance. This means that testing for accuracy differences per RT bin using an analysis of variance (an instance of the general linear model) may yield incorrect conclusions, because the assumptions of the statistical test(s) are not met.

In the present article, we will introduce a novel method of analyzing the accuracy–RT relationship, based on nonlinear logistic regression. The new method does not suffer from the issues discussed above, yet it has the flexibility to account for the most common patterns in accuracy–RT data. It captures the shape of the accuracy–RT relationship without the specific potential pitfalls described above. The method is model-free in the sense that it does not commit to a specific cognitive modeling framework, yet the parameters can be interpreted in light of a specific hypothesis about the accuracy–RT relationship in one’s experimental data.

## Methods and results

We highlight another approach for estimating the dependence of accuracy on RTs: nonlinear logistic regression. Logistic regression models aim to predict a categorical response variable using a continuous predictor variable. In the present case, this is binary accuracy (i.e., a correct or incorrect response), which is predicted by RT. Thus, the logistic regression model estimates the probability of a correct response based on a particular RT. Although a common assumption of logistic regression is the linear dependence of the response variable (correct or not) on the predictors, we propose a nonlinear transformation of the predictors to account for the typical patterns observed in the CAF literature. A first requirement of the nonlinear transformation is that it is nonmonotonic, meaning that the probability of a correct response can both decrease and increase. A second requirement is that the shape of the function is flexible enough that it can account for asymmetric response patterns. That is, the increase in the probability of a correct response with fast RTs is typically faster than the decrease with slower RTs. The shape of the patterns of faster errors is not necessarily the same as the shape on the decrease of slower errors (unlike in, e.g., a parabola). A third requirement is that the function can be parameterized in such a way that each parameter captures a specific qualitative property of the shape of a CAF, relating the behavioral phenomena expressed in CAFs to parameter-specific changes.

Following these requirements, we propose to model the probability of a correct response at a certain RT using Eq. 1:

1 $$ p\left( correct|t\right)=\frac{e^a}{e^a+{e}^{\left(b\left(t-d\right)+\frac{c}{t-d}\right)}} $$

**Fig. 2:**
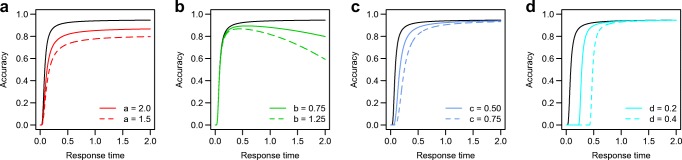
Different parameters have different effects on the shape of the function. (**a**) The *a* parameter defines an upper bound. (**b**) The *b* parameter defines the downward slope of the second segment of the function. (**c**) The *c* parameter defines the initial bend of the first segment of the function. (**d**) The *d* parameter defines a shift of the function. The black line in each panel is parameterized according to *a* = 3, *b* = 0, *c* = 0.25, *d* = 0. The colored lines deviate from this in one parameter, indicated by each legend.

Equation 1 is an instance of the logistic function parametrized by four parameters that together account for the full range of data patterns observed in the accuracy–RT relationship. It defines the probability of a correct response, *p*(correct | *t*), as a function of the RT *t.* Parameter *a* defines an asymptote that captures the maximum accuracy that is obtained (Fig. 2a). A lower *a* gives lower maximum accuracy. Parameter *b* defines the downward slope of the second segment of the curve (Fig. 2b). If *b* = 0, there is no downward section, but for all *b* > 0 the parameter value determines how steep the function declines after its peak value. Larger *b* results in a steeper slope and an increase in the number of slow errors. Parameter *c* defines the location of the peak value or bend point (Fig. 2c). The *d* parameter defines a shift of the curve over the *x*-axis (Fig. 2d). A positive value of *d* indicates that the curve is not defined for RTs smaller than *d*, which can be interpreted as a lower bound on the observed RTs: the lower limit of the accuracy–RT shape.

If *b* = 0 and *c* = 0, Eq. 1 reduces to $p\left(\mathrm{correct}|t\right)=\frac{e^a}{e^a+1}$. In this case, if *a* approaches infinity, the probability of a correct response approaches 1. If *a* = 0, the probability of a correct response is .5, with negative values for *a* yielding lower probabilities of a correct response. In this specific case, the probability of a correct response is also not predicted to change for different RTs, since the probability does not depend on *t* anymore.

If *c* = 0 but *b* is positive, the probability of a correct response is determined by *a* if *t* – *d* = 0 (i.e., at the fastest response), but declines for increasing RTs *t*. If *c* is positive as well, then the probability of a correct response first rises with increasing RTs, but then declines. In practical situations, this initial rise and eventual drop is the most typical pattern, and therefore we suggest constraining all parameters to the positive range when estimating their values on the basis of empirical data (see the Application section below).

### Parameter recovery

The goal of this analysis tool is to reach better conclusions about the accuracy–RT relationship, which will help support or reject hypotheses that researchers have about cognitive processes. For this purpose, it is crucial to study whether it is possible to identify the data-generating parameters in a sample of synthetic data (Anders, Alario, & Van Maanen, 2016; Miletić, Turner, Forstmann, & Van Maanen, 2017). If the data-generating parameters can be recovered reliably, then the parameters estimated from the data can be interpreted in support of a specific hypothesis.

To study this, we performed a parameter recovery study. The setup of the parameter recovery was a follows:

1. We defined the parameter space from which we sampled.
2. Data were generated according to one parameter vector.
3. We estimated the parameters of the logistic regression model for this new data set.
4. Steps 2 and 3 were repeated 1,000 times, to sample the full parameter space.

#### Parameter space

**Fig. 3:**
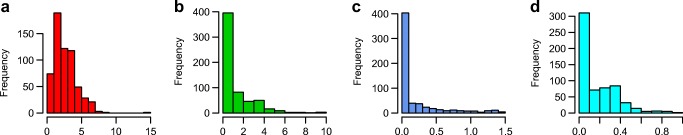
Distributions of parameters estimated from the data sets in Table 1.

To make sure that our parameter recovery involves reasonable parameter ranges, we estimated parameters for experimental data from ten experiments with multiple participants and conditions, for a total of 750 data sets (Table 1). The experiments included perceptual judgments, memory-based choice tasks, and a Simon experiment. Care was taken to include data sets that we hypothesized would affect every parameter, to ensure that critical cases were also included in the parameter space. In addition, the parameter space was constrained to positive values. The resulting distributions of parameters from which we sampled are shown in Fig. 3.

**Table 1:** Brief description of the experimental data sets fitted to obtain a sensible parameter space

Reference	Experiment	Conditions	Participants
Forstmann et al. (2010)	Perceptual judgment with response bias	5	17
Katsimpokis et al. (2018)	Expanded judgment with speed–accuracy trade off and response deadlines	4	24
Katsimpokis et al. (2018)	Perceptual judgment with speed–accuracy trade off and response deadlines	4	24
Maass et al. (2016)	Delayed expanded judgment task	8	24
Maass et al. (2016)	Expanded judgment task with reward	4	24
Maass et al. (2016)	Expanded judgment task with response deadline	3	21
Van Campen et al. (2014)	Simon task	2	10
Wagenmakers et al. (2008)	Lexical decision task with response bias	2	18
Wagenmakers et al. (2008)	Lexical decision task with speed–accuracy trade-off	2	17

#### Data generation

On each of 1,000 iterations, we randomly sampled a set of parameters estimated from one of the 750 data sets. We computed the predicted accuracy for the range [50 ms–5 s], discretized by steps of 5 ms, and generated binary response data by performing exactly one Bernoulli trial for each discrete time step in the range, with the probability of a positive outcome set at the predicted probability of the logistic regression model (for this set of parameters). This procedure resulted in 1,000 simulated data sets consisting of response-RT pairs that could be subsequently used to estimate parameters.

#### Parameter estimation

The parameters of the so-generated data sets were estimated using SIMPLEX optimization of the squared residuals (Nelder & Mead, 1965). The optimizer minimized the weighted sum of the squared errors using the ordinary least squares procedure. As starting values of the SIMPLEX search, we used the mean values of the parameters in the parameter space. This procedure resulted in the set of four parameters for the simulated data that had the lowest squared error.

**Fig. 4:**
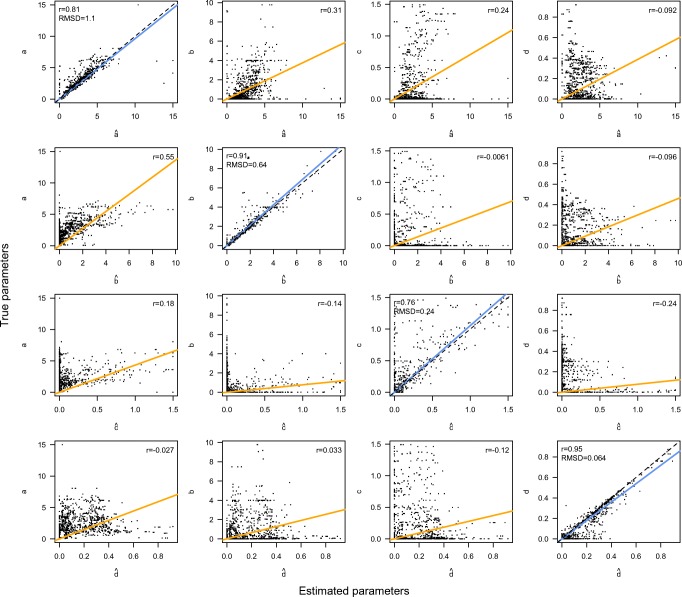
Results of the parameter recovery. See the text for details.

Figure 4 displays that the parameters were reliably recovered. The values on the *y*-axes indicate the parameters that were used to generate the data sets, and the values on the *x*-axis indicate the estimated parameters The panels on the diagonal display how well the parameters were recovered. A perfect recovery would mean that all true estimated pairs would lie on the dashed line. The blue regression lines indicate the actual relationship between true and estimated parameters, which closely follows the perfect line, indicating that on average the logistic regression model nicely recovered the true parameters. The diagonal panels also display the correlations between the true and estimated parameters, as well as the root mean squared deviations (RMSD) between the true and estimated parameters. These measures indicate that the parameter recovery of the logistic regression model was satisfactory.

Off the diagonal, the relation of the parameter estimates to the *other* true parameters is displayed, providing a measure of trade-off between the parameter estimates. The correlations between the parameter estimates and the other true parameters are clearly below the diagonal, although they are quite high for the *a* and *b* parameters. The interpretation of these correlations is that effects in the data that are explained by the *b* parameter are also partially explained by the *a* parameter. This is potentially a consequence of a quite high correlation in the parameter spaces between the *a* and *b* parameters of *r* = .59: Intuitively, this makes sense: A higher asymptote indeed allows for a greater negative slope in the second half of the accuracy–RT data.

#### Recovery in smaller data sets

The first parameter recovery studies had a highly idealized scenario with many observations equally spaced across the full RT range. However, in practice, data typically have fewer observations and are not evenly distributed. To show that our method is resilient to more realistic data patterns, we performed an additional parameter recovery, in which we included only 200 responses per simulated data set. The simulated RT data were generated according to a shifted Wald distribution function, which is a common description of RT distributions (cf. Anders et al., 2016). The mean and shape parameters of the shifted Wald function were uniformly sampled across simulations (between 0.5 and 2 and between 2 and 4, respectively). This parameter recovery revealed that, although slightly less accurate, the model still showed strong correlations between the true and estimated parameters (Table 2). This means that for experiments with around 200 trials, the parameters of the logistic regression model can be reliably interpreted to study the accuracy–RT relationship.

**Table 2:** Results of Parameter Recovery 2

True Parameter	*r*	RMSD
*a*	.63	2.0
*b*	.89	0.8
*c*	.53	0.3
*d*	.81	0.1

See the text for details.

### Application to standard data sets

In this section, we illustrate how conclusions can be drawn by the application of the non-linear logistic function to the accuracy–RT relationship. In Example 1 the model was fit to data of a lexical decision task in which participants were cued to apply a speed–accuracy trade-off (Wagenmakers, Ratcliff, Gomez, & McKoon, 2008). Here we expected the *a* parameter to vary across conditions (for reasons that will be specified below). Example 2 pertains to an experiment in which time pressure was experimentally manipulated (Van Maanen, Fontanesi, Hawkins, & Forstmann, 2016), and we expected differences in the *b* parameter. In Example 3 the model was fit to data from a Simon task (Van Campen et al., 2014), and we expected the *c* parameter to vary across conditions. Because the *d* parameter is comparable to a nondecision time parameter in sequential-sampling models and is often considered uninformative and only used to better fit the data, we will not provide an additional application example for this parameter.

#### Cue-induced speed accuracy trade-off

*Cue-induced speed–accuracy trade-off* refers to the often observed result that when participants are cued to respond fast, they can only increase their speed of responding at the expense of their response accuracy (Bogacz et al., 2010; Heitz, 2014; Wickelgren, 1977). A common interpretation of this finding is that in many situations, participants contemplate their actions until they reach a specific level of certainty about the planned action, and then commit to that action. When they are pressed for speed, this level of certainty is hypothesized to be less. One very prominent class of mathematical models (a simple diffusion decision model that assumes no between-trial variability in the rate of evidence accumulation; Ratcliff, 1978) suggests that although participants indeed trade response accuracy for response speed in this scenario, there is no dependence between the probability of a correct response and response speed *within each condition.* This suggests that fitting the CAF should result in a lower *a* parameter under cue-induced speed stress (see also Fig. 2a), but not differences between conditions for the remaining parameters.

In the lexical decision task of Wagenmakers et al. (2008), participants were asked to indicate with a button press whether or not a letter string presented on a computer screen was a valid English language word. Each of 17 participants contributed 960 responses while it was stressed that being accurate was more important than being fast, and 960 responses while the opposite instruction was provided: The speed of responding was more important than accuracy. In half of the trials, valid words were presented, and in the remaining trials items were presented that resembled words but in which one or a few characters were adjusted to create an invalid word (for details about the experimental design, we refer the reader to Wagenmakers et al., 2008). The word and nonword trials are collapsed for the purposes of the present analysis.

We fit the CAF separately to the individual speed–accuracy conditions and participants. The parameters of the CAF were optimized using SIMPLEX optimization (Nelder & Mead, 1965), with reasonable starting points (*a* = 3, *b* = 0.1, *c* = 0.01, *d* = 0.1) that did not differ across participants and conditions. All parameters were bound within the (0, Inf) range (i.e., parameter estimates could not become negative).

Because of floor effects on the possible parameter estimates, we log-transformed the *b* and *c* parameters before performing statistical analysis. Paired *t* tests indicated a significant difference in the estimates of *a* [*t*(16) = 3.16, *p* = .006], as well as a significant difference in the log-transformed estimates of *c* [*t*(16) = – 2.65, *p* = .017]. The other two parameters did not differ significantly [*t* values < 1.1; a test on the nontransformed *b* estimate also did not reach significance: *t*(16) = 1.4, *p* = .16].

**Fig. 5:**
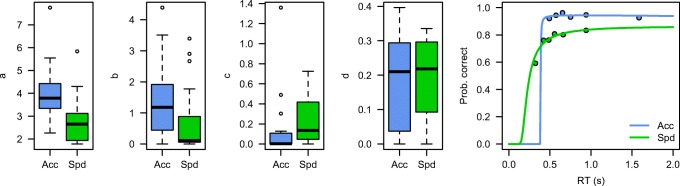
Parameter estimates of the speed–accuracy trade-off data set. The right panel shows a representative participant, with the predicted nonlinear logistic CAFs overlaid on the bin-based accuracy estimates. Acc: accuracy instructions; Spd: speed instructions.

Thus, as predicted, the cue-induced speed–accuracy instruction provided in this experiment was reflected in the *a* parameter, yielding CAFs that asymptoted at different levels (Fig. 5). In addition, it seems that in this data set, part of the speed–accuracy trade-off behavior can be explained by a higher proportion of fast guesses, as indicated by the higher estimate of *c* for speed-instructed trials (Dutilh, Wagenmakers, Visser, & Van der Maas, 2011; Schneider & Anderson, 2012; but see Van Maanen, 2016).

#### Urgency

Recent work has suggested that an error rate that increases with RT is a signature of time pressure or urgency (Hanks et al., 2014; Murphy et al., 2016; Thura & Cisek, 2016). That is, if participants have to make decisions under time pressure (e.g., before a certain time limit has been reached, but not if they are simply cued to be fast, as above; cf. Katsimpokis et al., 2018), then they might make more mistakes the longer the decision process takes, resulting in a negative slope of the CAF, which is expressed by the *b* parameter (see also Fig. 2b). An increase in *b* therefore may reflect a decision boundary that decreases over time (Frazier & Yu, 2008; Malhotra, Leslie, Ludwig, & Bogacz, 2017).

Van Maanen and colleagues (Van Maanen et al. 2016) explored this line of reasoning using an expanded judgment paradigm, in which participants were asked to make a choice about a noisy stimulus that slowly built up on the screen. The researchers manipulated the speed of the buildup, thereby inducing time pressure effects without experimentally manipulating the choice difficulty. Specifically, the interval between consecutive updates of the stimulus was either 200 or 400 ms, meaning that the same amount of information about the stimulus was presented in half the time in one condition as compared to the other condition (on average, across trials). Van Maanen et al. (2016) found that when the stimulus updated quickly, participants felt pressed for time and made decisions based on less information on the screen. Moreover, a negative relation was found between RT and the amount of information, similarly indicating that participants felt time pressure during a trial. A nonnegative *b* parameter in the present analysis would be conceptually similar to these findings. Here we present the data from their Experiment 2, which was conducted while participants were in an MRI scanner (for details of the experimental design, we refer the reader to Van Maanen et al., 2016).

**Fig. 6:**
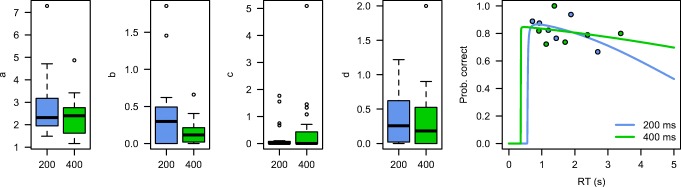
Parameter estimates of the time pressure data set. The right panel shows a representative participant, with the predicted nonlinear logistic CAFs overlaid on the bin-based accuracy estimates.

Figure 6 presents the distributions of the estimated parameters. Because of floor effects on the possible parameter estimates, we again log-transformed the *c* parameter before performing statistical analysis. In this case, we judged this additional step unnecessary for the *b* parameter, because the median RTs were sufficiently above the floor value of 0. Although the parameter estimates did not differ significantly [at an alpha level of .05, *t*(19) = 1.90, *p* = .07], we believe that it is interesting that the direction of the effect was consistent with the previous results and with the previous analyses of these data (Van Maanen et al., 2016). Also note that no other parameter showed an effect [*a*: *t*(19) = 1.28, *p* = .21; with respect to the *c* and *d* parameters, *t* values < 1].

#### Response capture

In certain experimental paradigms, the stimuli are such that they seem to engage an automatic process, resulting in very fast but error-prone responses. This process, sometimes referred to as *response capture*, occurs in the Simon task. In this task, participants are asked to indicate with a left or right button press whether a stimulus has a specific color. Because the stimuli appear on the left or right side of the screen, this often elicits a fast response with the hand on the same side as the stimulus. If the stimulus location is congruent with the (correct) response hand, indicating that an automatic button press yields a correct outcome, there is no decrease in accuracy for fast responses. For incongruent trials, however, this leads to a large proportion of errors, decreasing the overall accuracy for fast responses (Forstmann et al., 2008; Ridderinkhof, 2002; Van Campen et al., 2014; Van Campen et al., 2018; Van den Wildenberg et al., 2010). Although the standard method in the field has been to compute the proportion of errors in the first RT bin, as we outlined above, a novel methodology has recently become available that addresses the specific question of response capture (Servant, Gajdos, & Davranche, 2018).

We predict that response capture should be visible in the *c* parameter of the CAF function, since that parameter decreases the accuracy in the initial segment of the curve for incongruent trials, which would be consistent with the higher fast-error rate associated with response capture (see also Fig. 2c).

Van Campen et al. (2014) did a fairly standard version of the Simon task, in which ten participants had to respond with a right or left button press (counterbalanced across participants) to whether a circle that appeared on the screen was green or blue. During the experiment, single-pulse transcranial magnetic stimulation (TMS) was administered in order to obtain indices of corticospinal excitability. Crucially for the present purpose, the single-pulse TMS did not disrupt behavior in any way (for details of the experimental design, we refer the reader to Van Campen et al., 2014).

**Fig. 7:**
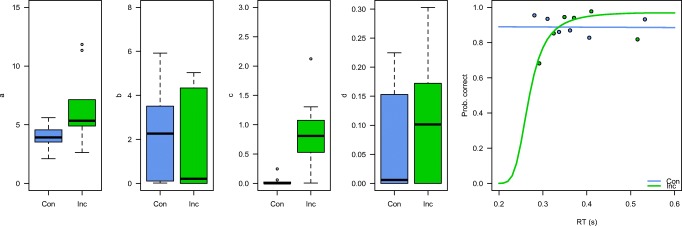
Parameter estimates of the Simon task data set. The right panel shows a representative participant, with the predicted nonlinear logistic CAFs overlaid on the bin-based accuracy estimates. Con: stimulus location and response hand are congruent; Inc: stimulus location and response hand are incongruent.

Figure 7 displays the distributions of parameter estimates, as well as the typical CAFs for one representative participant. Because of floor effects on the possible parameter estimates, we again used log-transformation of the *b* and *c* parameters before performing the statistical analyses. Paired *t* tests resulted in a significant difference in the *c* parameter [*t*(9) = 5.9, *p* < .001], suggesting that participants indeed had more fast incorrect responses in the incongruent than in the congruent condition. In addition, the *a* parameter differed between conditions [*t*(9) = 2.7, *p* = .02], indicating that the asymptote of the CAF was higher for the incongruent than for the congruent condition. The *b* and *d* parameters did not differ between conditions (*t* values < 1.6).

### Another inferential method: Model comparison

Another method of inference is to compare how well models balance their goodness of fit and their flexibility to account for data (Pitt & Myung, 2002). That is, if a model has many parameters, in many cases it can also account for many different patterns in the data. Thus, a model that is overly flexible in this way might overfit the data, and so not generalize to other data sets. In those cases, a simpler model might account for the data almost as well, by explaining the true effects in the data but not the noise that is inherent in a specific data set.

Many methods exist to formally compare the goodness of fit of two models with different levels of flexibility. One typical method for this model comparison is the Akaike information criterion (AIC; Akaike, 1974), which corrects the goodness of fit for the number of free parameters of each model. We illustrate this method here on the data of Van Maanen et al. (2016), to illustrate its use in the context of CAFs and show that there is evidence that the *c* parameter is effectively zero in this data set, as was already suggested by Fig. 6. In this case, we wanted to test whether a nonzero *c* parameter explained a significant proportion of the explained variance. Therefore, we compared the *full* model discussed above, in which all parameters were estimated, with a *reduced* model in which we forced the *c* parameter to be *c* = 0. Consequently, the reduced model had fewer free parameters, and one could compare the balance of the flexibility of the models with respect to their goodness of fit. If the reduced model fit almost as well as the more complex full model, even though it was less flexible, it would be considered the better model.

Because we applied least-squares parameter estimation, it was possible to compute AIC values through the residual sum of squares of the models (RSS; Burnham & Anderson, 2002). For each participant and condition, the AIC was computed according to the formula AIC=2*k*−*n*ln(RSS), with *n* being the number of observations per cell, and *k* the number of free parameters, which was *k* = 8 for the full model and *k* = 6 for the reduced model. Comparison of the AIC values obtained in this way revealed that in 92.5% of the cases, the reduced model was preferred over the full model (for 90% of the participants in the 200-ms condition, and 95% of the participants in the 400-ms condition). Akaike weights (Wagenmakers & Farrell, 2004) showed that the reduced model was about twice as likely to be correct as the full model [averaged AIC_w_(Full) = .32; averaged AIC_w_(Reduced) = .68]. Thus, although the difference between the AIC values was not big (as revealed by the Akaike weights), the reduced model was consistently preferred over the full model, leading to the inference that there were no specifically fast incorrect responses in this data set. This conclusion thus also corroborates our previous analysis that showed no significant difference between the *c*-parameters in the Van Maanen et al. (2016) data, but with the stronger claim that these parameters were in fact equal to 0.

## Discussion

There are many researcher degrees of freedom in the typical methods for identifying relationships between accuracy and RTs, and potential pitfalls exist in interpretation due to the chosen strategy. For this reason, we introduced a novel approach to computing CAFs, which is based on nonlinear logistic regression and solves the problem of choosing the exact amount of RT bins. The logistic regression model assumes a flexible functional form that accounts for the typical patterns in the data, and using least squares regression the best fitting parameters can be estimated. In simulation and in example applications, we showed that the estimated parameters are accurate and that meaningful inferences can be made.

It is pertinent to finish the discussion of this approach by stressing its limitations. In our view, three important limitations warrant discussion. First, it is worth mentioning that the logistic regression model is only applicable to binary outcomes (typically correct/incorrect). For most application domains, however, this is not problematic.

Second, although it is an improvement over current practices, the new method still allows for some researcher degrees of freedom. Specifically, as with any mathematical model, there are multiple ways of drawing inferences. We have focused here on performing statistical tests on the estimated parameters. Another method that we discussed is to compare the fit quality and the flexibility of two (or more) models that implement different theoretical assumptions about the data. We illustrated this approach with a model that did not allow for specifically fast incorrect responses, and one that did, and compared these using AICs. Other methods are also available, potentially leading to different inferential outcomes (Dutilh et al., 2018).

A related issue is that the standard statistical tests that we chose to perform on the parameters have relatively strong assumptions. Violating these assumptions might increase the chance of an incorrect inference if this were not properly controlled for. In the case of the logistic regression model, the lower bound on the parameter space may result in non-Gaussian distributions of the parameters. Here we chose to apply a logarithmic transformation of the data to obtain more Gaussian-like distributions when this was the case, but this is a degree of freedom that is allowed to the researcher, as well (Gelman & Loken, 2014).

A third important limitation is that the present setup of the model does not allow for the inclusion of random effects, which may be pertinent in application domains where stimuli differ from trial to trial (Anders, Oravecz, & Alario, 2018). Here we chose to estimate the model parameters independently for each condition and to do inference on the group level in a second stage, but in situations with small sample sizes or large item effects, an analysis that included (crossed) random effects might increase power (Baayen, Davidson, & Bates, 2008).

### Conclusion

The present article has introduced a new method for analyzing conditional accuracy in a principled, model-free way. The method alleviates some of the problems associated with RT binning to obtain different accuracy levels per RT bin. Specifically, the researcher degrees of freedom are reduced. Using this method, we can reliably identify the presence of fast or slow errors, which may be beneficial in many domains of cognitive psychology in which the relationship between responses and RTs is of theoretical importance.

#### Author note

D.K. is supported by the Onassis Foundation Scholarship program for Helenes.
